# The changing relationship between marriage and childbearing in Hong Kong

**DOI:** 10.1371/journal.pone.0194948

**Published:** 2018-03-29

**Authors:** Stuart Gietel-Basten, Georgia Verropoulou

**Affiliations:** 1 Division of Social Science and Division of Public Policy, The Hong Kong University of Science and Technology, Clear Water Bay, Kowloon, Hong Kong SAR, People’s Republic of China; 2 Department of Statistics and Insurance Science, University of Piraeus, Piraeus, Greece; University of West London, UNITED KINGDOM

## Abstract

Births outside marriage (BoM) account for around 15% of all births globally. However, the distribution around the world is very uneven, as are cultural and political attitudes towards them. Studies from East Asia have shown that the percentage of such births is very low, with only modest increases in recent years. The orthodox demographic view holds that the maintenance of conservative views around the relationship between marriage and childbearing can play a role in keeping fertility low. Prenuptial pregnancies (PNP) (where births occur within eight months of marriage) have been identified as a growing phenomenon in Japan, possibly being an ‘alternative’ Asian pathway to family formation. As yet, no comprehensive statistical analysis of the trends of BoM or PNP has been performed for Hong Kong. Using a comprehensive microdata set of birth registration in Hong Kong from 1984–2015 (N = 1,680,831) we provide evidence of recent trends in such ‘alternative pathways’ to family formation and examine predictors through regression analysis. Our results indicate, in common with elsewhere in East Asia, low overall period rates of either BoM or PNP (although the latter has risen notably in recent years). While more recent birth cohorts exhibit higher prevalence of such births, their incomplete nature and higher expected propensity suggests that the figures are exaggerated. In our regression analysis, we find that lower educational attainment is a strong predictor of both BoM and PNP, suggesting that a bifurcation of experience may be occurring. This adds further evidence to the theory that the maintenance of traditional family formation systems in the context of revolutionised educational and work opportunities for women mean that the opportunity costs of the ‘marriage package’ become too high. Current disparities in rights and privileges between married and unmarried parents–and especially their children–means that targeted family planning services and support for vulnerable families are policy priorities.

## Introduction

In 2016, 15% of all of the births in the world occurred outside of marriage [1]. However, this figure masks an enormous variation across countries. For example, the average percentage of births outside marriage (BoM) in the EU and among OECD countries is around 40%; while in Chile, Iceland and Costa Rica more than 65% of births occur outside marriage. On the other hand, less than 3% of births in Japan, South Korea and Turkey occur outside of wedlock [2]. Figures for China and India are similarly low [1]. Over time, there is also heterogeneity in patterns of change. While Sweden has one of the highest percentages of BoM at 54.6% in 2014, this figure has remained relatively unchanged since the late 1980s. Some countries–especially in Southern and Eastern Europe–have seen dramatic increases over the same period. In Cyprus, less than 1% of births occurred outside of marriage in the early 1980s; they now account for around 20%. Over the same period in Bulgaria, the percentage of BoM rose from around 20% to almost 60% [2]. In reality, there are many possible configurations as far as the relationship between marriage and childbearing is concerned: divorce, remarriage, co-habitation, marriage after birth, and so on [3–6]. Similarly, unthinking comparisons across countries can also obscure important cultural differences in the institution and nature of marriage itself.

Such changes in the relationship between birth and marriage are of cultural, sociological and demographic importance. Culturally, the reshaping of the family unit over time and space–at different paces–represents one of the key shifts in contemporary social relations. This has been represented in both positive and negative lights by various types of commentators, representing the liberation of ‘freedom from status fate’ and the potential to ‘design one’s own autobiography’ [7] through to the favoured theme of many conservatives critics of the breakdown of the ‘conventional family’ (defined as children born within marriage) being associated with a wide range of social problems [8] (despite the fact that co-habitation and consensual unions have a long history in various parts of the world [9]). Much public discourse, as well as academic literature, often takes such a bifurcated view of births outside of marriage. Within Second Demographic Transition Theory, for example, the weakened link between childbearing and marriage is most closely linked to a Maslowian drift and a rise in individuality and self-actualization associated with improved education and the demographic ‘vanguard’ [10]. It is, however, often difficult to reconcile this ‘vanguard’ approach with the high rates of births outside of marriage found in population subgroups in OECD countries who are often characterized as poorer and where children have, comparatively, less successful outcomes [11]. Indeed, many countries still give preferential treatment to married couples with children: the United States offers over 1000 benefits for married couples as compared to singles, for example [1].

The subject of BoM has long been of interest to demographers for both empirical and theoretical reasons. It is the case that countries with very low rates of BoM and higher levels of development are also characterised by very low levels of overall fertility. The linkages seem clear. The marriage ‘package’ in such countries represents a particular type of strict institution seemingly at odds with ever more ‘individualized’ behaviour [7]—especially in East Asian societies [12–14] which are characterised by very low fertility rates [15], and where there is an ‘incomplete gender revolution’ of a mismatch of female opportunities in the labour market and restrictive, traditional domestic expectations [16–18]. Also, the actual cost of the wedding itself is often presented as a disincentive [19]. Taken together with a Maslowian drift interpretation, Lesthaeghe [20] suggests that the shift from a world which revolved around the child to one which placed ever more importance on the adult dyad–the ‘king couple’ in Aries’ terms–an increase in the minimal standards of union/marriage quality naturally ensued. Taken together, it might be assumed that without the reform of marriage as an institution, or a sizable shift in the number of births outside of marriage, the link to lower fertility may stay constant [21,22].

As already noted, East Asian societies are characterised by very low rates of BoM. In the low fertility settings of South Korea, Taiwan, China, and Japan these figures are generally below 5% [1,2,22,23]. The prevalence of traditional attitudes regarding the relationship between marriage and childbearing in these societies is well documented in the literature [24,25]. In numerous settings, stigma and discrimination against children born out of wedlock is rife [26]; as well as policies actively designed to discourage such births [27]. While studies have suggested that attitudes against BoM are not strong per se [28,29], marriage is still seen to be a defining and clear step in the life course.

According to recent surveys from Japan, Taiwan and China, between 20–30% of adults born in the 1970s cohabited at some point in their lives [25]. However, rather than an alternative to marriage [9], these are generally rather short periods of time, often seen as a precursor to marriage (or as a ‘test run’) [25]. In 2010, for example, only 2% of unmarried Japanese females aged 25–29 were in a cohabiting union [30]. In this context, there have been numerous studies of so-called ‘prenuptial’ or ‘bridal’ pregnancy–especially in Japan–where births occur within eight months of marriage. Hertog and Iwasawa [31] find that 90% of premarital pregnancies (PNPs) in Japan result in births within marriage–compared to just 10% in the United States. Elsewhere, Raymo and Iawasawa [32] suggest that increases in PNP in Japan over recent decades were primarily concentrated among women without postsecondary education. This, again, suggests that view of a professional ‘vanguard’ pushing the rise in ‘non-traditional births’ might be misplaced. PNPs, therefore, represent an important sociological phenomenon: a potentially unique Asian alternative to the more traditional trajectory of marriage, then co-residence, then conception.

The Special Administrative Region of Hong Kong has a population of 7.3 million and had, in 2016, one of the lowest total fertility rates in the world at 1.20 [33]. As elsewhere in Asia, cohabiting couples enjoy many fewer rights than married couples including tax, pension, medical and public housing benefits [34], as well as fewer legal rights [35].

Again, in common with elsewhere, surveys suggest that cohabitation is a relatively short-term phenomenon, often as a precursor to marriage [36]. While social attitudes towards BoM and cohabitation are changing–especially among the young [37,38]—this quote from a recent discussion in the Legislative Council over whether parental leave should be granted to unmarried fathers suggests the prevalence of a traditional view (emphasis added):

The community is broadly of the view that PL [Parental Leave] should be accorded to “husbands”, i.e. legally married males, only. However, failing to grant PL for childbirths outside of marriage might constitute discrimination on grounds of marital status and family status under the Sex Discrimination Ordinance (Cap. 480) and the Family Status Discrimination Ordinance (Cap. 527) respectively and as such might be in breach of these Ordinances [39].

While a number of studies have explored the context of fertility decline in Hong Kong [21,40–42] as well as patterns of marriage [43–45] and divorce [46] and the links to individualization [12], as yet there has been no comprehensive statistical analysis of trends and predictors of either BoM or prenuptial pregnancies in Hong Kong. These studies have only considered the issue in passing. Yip [21], for example, in making the link between low rates of BoM and low fertility notes that ‘It is very unlikely to see a rebound of fertility among the Hong Kong women in the near future if there is no increase in marriages or births outside wed-lock’. Raymo et al’s recently published magisterial recent review of marriage and the family in East Asia covering such themes as BoM and PNPs; however, discussion of the circumstances in Hong Kong were omitted from that analysis [25]. In this paper, then, we attempt to fill this gap in the literature by examining recent trends in the changing relationship between birth and marriage in Hong Kong, and exploring their significance for the demographic, policy and social situation in the territory.

## Materials and methods

### Data

For the purposes of this analysis, vital registration microdata on births in Hong Kong were obtained by the Hong Kong Census and Statistics Department for the period 1984 to 2015. Births occurring to transient Chinese women (i.e. women originating in the Mainland, who are resident in Hong Kong for less than a year before giving birth) are excluded from the analysis [47]. In the overall period, there were 1,682,155 births recorded; 1,324 of these (0.08%) had missing information on the marital status of the parents and were dropped. As such, the analysis on BoM refers to 1,680,831 observations.

PNPs were defined as births occurring within eight months of marriage to allow for comparability with other studies [32]. However, as these births may include a small proportion of viable premature foetuses, a second definition was also used, referring to births occurring within six months of marriage, enabling us thus to check the robustness of our findings based on the main definition. To identify PNPs complete information on the date of the marriage of the parents was required, which became available from 1988 onwards. Overall, 1,399,850 births were registered in the period 1988–2015 (excluding transient Mainland births); 87,064 of these were BoM and thus, were not included. Of the remaining 1,312,786 births 8,797 (0.67%) had missing information on the dates of marriage and were also excluded from the analysis. Hence, the analysis about PNPs refers to 1,303,989 births.

### Statistical analysis and variables of interest

Apart from the descriptive findings, referring first to trends of BoM and PNP over time and by birth cohort of the mother and second to the characteristics of mothers having a BoM or a PNP, logistic regression was used to identify predictors. Two models were employed; one for births out of the wedlock and a second one for prenuptial pregnancies. In both cases models were run by birth cohort of the mother; further, all models control for age of the mother at the birth (in years) and year of birth of the child. It should be noted here that women born before 1970 would not have completed their reproductive careers by 2015; hence, findings for these women should be treated with caution. However, findings referring to women born earlier are not affected by such bias.

The main explanatory variable is maternal educational attainment which includes five categories: tertiary (degree), tertiary (non-degree), secondary, primary and no schooling/kindergarten. In this instance, tertiary (degree) was chosen as reference category. Analysis was performed using STATA 13.

## Results

**Fig 1:**
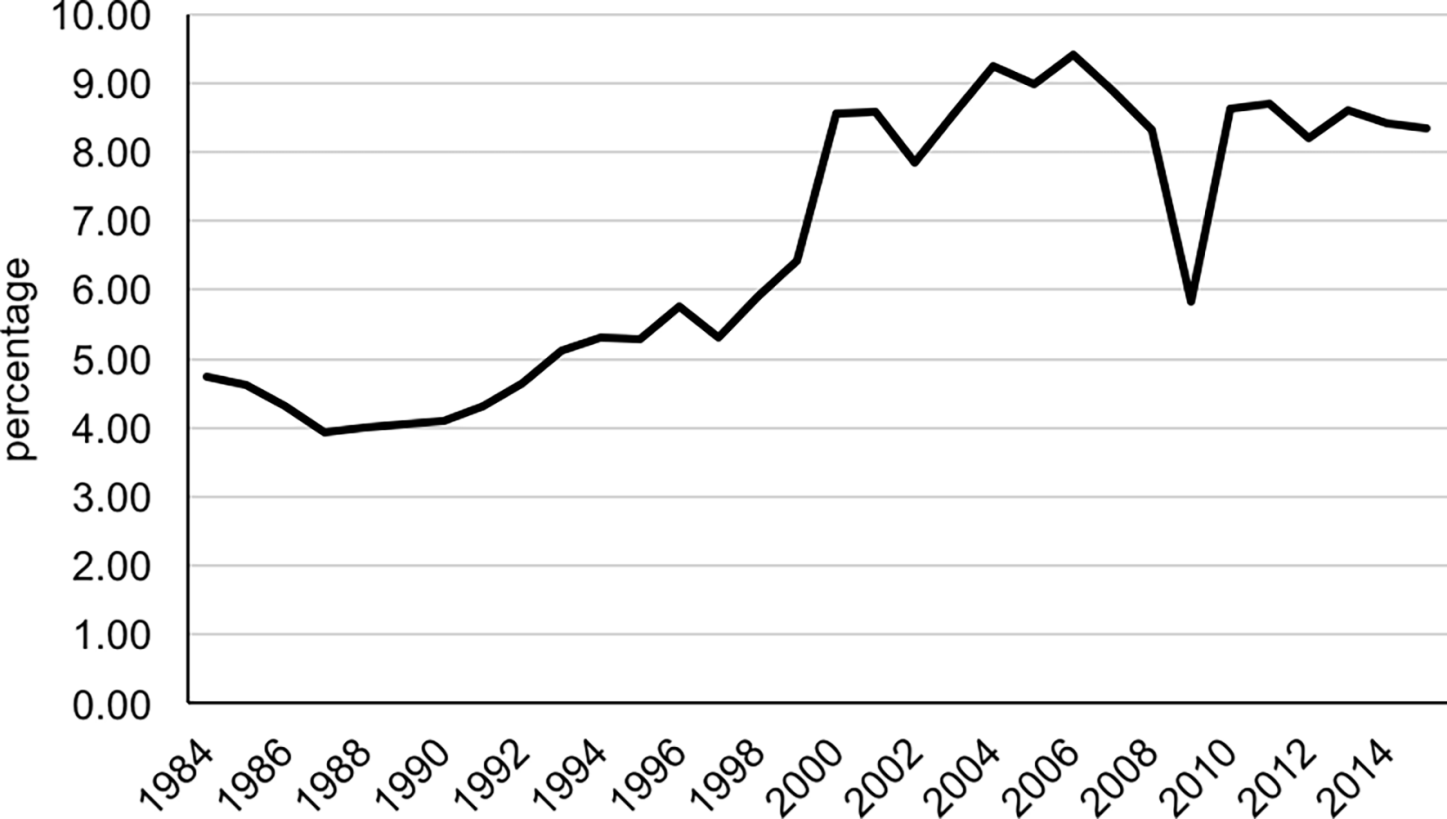
Extramarital births by year of birth, Hong Kong (1984–2015).

**Fig 2:**
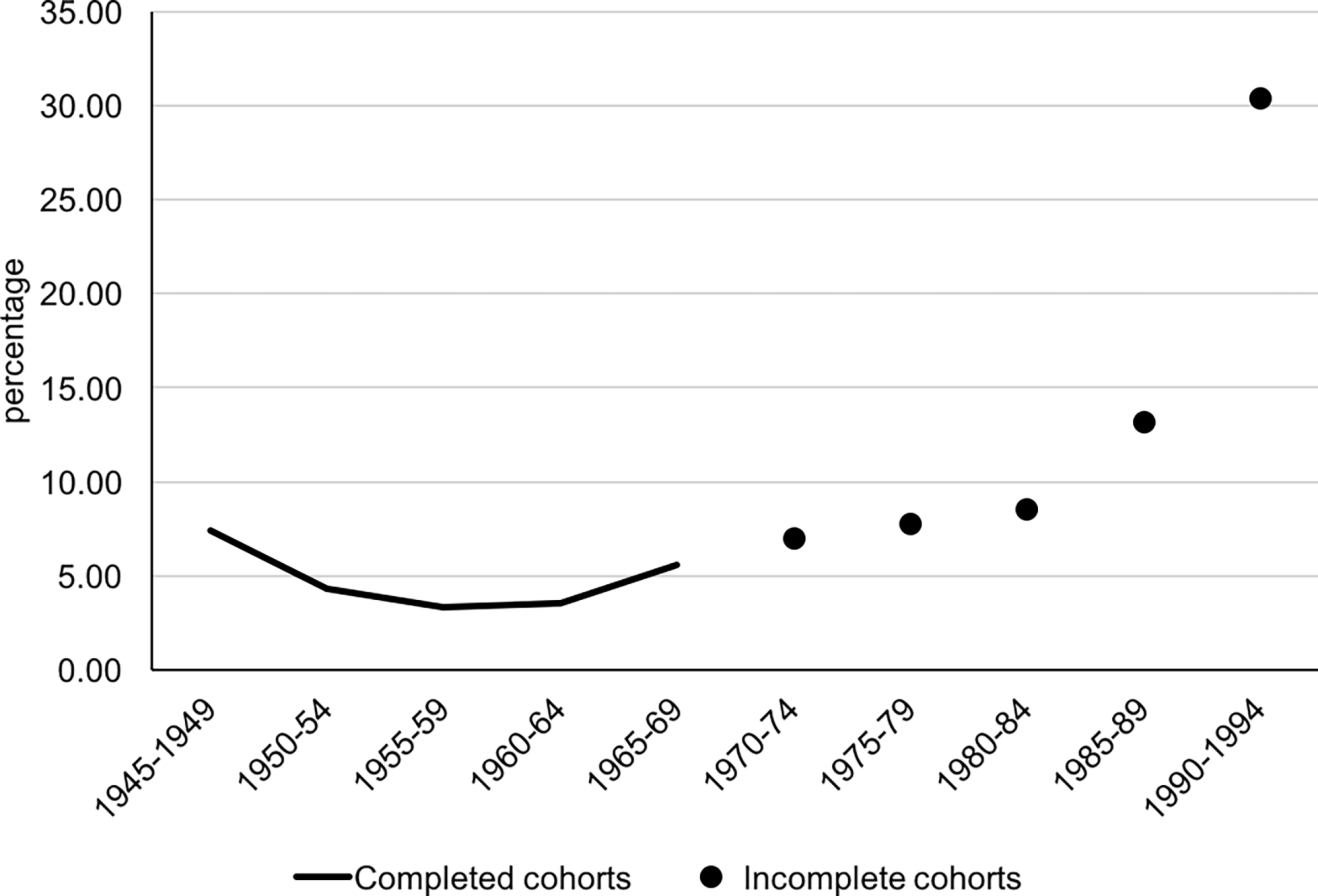
Extramarital births by birth cohort of the mother, Hong Kong (1945–1994).

Fig 1 shows the percentage of BoM by year of birth of the child for the period 1984–2015. This percentage fluctuated around 4–6% in the period 1984–1998 then rose to 8–9% by 2000 but remained rather constant thereafter. Regarding the percentage of BoM by birth cohort of the mother (Fig 2), it stood at 7.4% for cohorts born before 1950, reached a low (3.3%) among women born in 1955–59 and exhibited an increasing trend thereafter, reaching 8.6% for the 1980–84 cohort and 30.4% for women born in 1990 or later. It should be noted however, that only women born before 1970 had reached the end of their reproductive lives by 2015; for instance, women born in 1990 or later were aged 25 or less. Furthermore, a very high proportion of these BoM occurred within a cohabiting union, especially for women born before 1960 (95%). Even for women born in 1985–90 the respective proportion is 80% and for the 1990+ cohort it is 75%.

Table 1 shows descriptive statistics for women having a BoM in comparison to women having a birth within marriage. Considering women who have completed their reproductive careers, mean age at birth decreases marginally for both groups from about 32 for those born in the late 1950s to 29 years for those born a decade later. (Note that the very high mean ages of childbearing for the cohorts born before 1950 are due to the fact that only later births in these older cohorts will be captured by our dataset). Whereas mean age at birth of women having a BoM was higher for cohorts born before 1965 by about a year, this is reversed for women born thereafter, with the gap widening between successive cohorts, reaching about 3 years for women born after 1980. Hence, among younger cohorts there seems to be a tendency of having BoMs at younger ages. Regarding education, women born before 1960 having a birth within marriage have somewhat higher qualifications compared to mothers having a BoM. However, for cohorts born after 1965 and especially after 1975 the opposite seems the case though the difference is minor; a higher proportion of young women with a BoM have completed secondary education while a higher proportion of women with a birth within marriage have no schooling or have completed primary education.

**Table 1:** Descriptive statistics by birth cohort of the mother for women with or without an extramarital birth: Hong Kong.

						*INCOMPLETE BIRTH COHORTS*				
	<1950	1950–54	1955–59	1960–64	1965–69	1970–74	1975–79	1980–84	1985–89	1990+
**Women having a BoM**										
**Mean age at birth**	38.87	35.02	32.00	30.15	29.28	29.58	28.28	26.05	22.95	19.52
**Maternal education (%)**										
**Tertiary degree**	2.01	3.37	2.75	2.10	0.85	0.30	0.14	0.16	0.05	0.06
**Tertiary non-degree**	1.73	3.01	5.39	6.64	5.92	5.82	4.97	3.62	2.21	1.20
**Secondary**	39.78	48.00	56.51	68.70	76.79	79.70	81.14	83.33	86.34	91.35
**Primary**	46.26	39.83	31.31	17.81	8.82	6.10	5.25	5.67	5.79	5.20
**No schooling/kindergarten**	10.22	5.79	4.03	4.74	7.62	8.08	8.50	7.22	5.62	2.19
**Women having a Birth within Marriage**										
**Mean age at birth**	37.94	33.97	30.82	29.40	29.76	31.01	30.98	29.12	25.66	21.60
**Maternal education (%)**										
**Tertiary degree**	5.43	5.34	3.88	2.32	1.02	0.41	0.29	0.17	0.18	0.10
**Tertiary non-degree**	3.57	4.92	5.63	6.13	5.96	6.05	3.93	2.08	1.86	1.45
**Secondary**	46.61	52.38	58.39	67.79	71.42	63.26	57.28	58.84	69.50	83.57
**Primary**	38.90	33.97	29.01	18.82	10.26	8.29	9.18	10.87	11.39	9.66
**No schooling/kindergarten**	5.49	3.39	3.09	4.93	11.34	21.98	29.31	28.04	17.06	5.23

Table 2 shows Odds Ratios (ORs) based on logistic regression models exploring predictors of BoM. For the cohorts of women born before 1960 the chances of having a BoM exhibit an increasing likelihood for older women; this trend, however reverses for younger cohorts. On the other hand, lower educational attainment than having obtained a tertiary degree is significantly associated with higher chances of such a birth, especially for younger cohorts. Hence, when controlling for age of the mother at birth it would seem that this phenomenon is more widespread among women of lower educational status.

**Table 2:** Odds Ratios based on Logistic Regression Models assessing predictors of an extramarital birth by birth cohort of the mother: Hong Kong.

						*INCOMPLETE BIRTH COHORTS*				
	<1950	1950–54	1955–59	1960–64	1965–69	1970–74	1975–79	1980–84	1985–89	1990+
**Predictors**										
**Year of birth**	1.012	1.016	1.009	1.088**	1.095**	1.047**	1.011	0.968**	0.940**	0.978
**Age of the mother at birth**	1.099**	1.103**	1.075**	0.954**	0.900**	0.926**	0.912**	0.888**	0.814**	0.680**
**Maternal education (ref cat: tertiary degree)**										
**Tertiary non-degree**	1.312	0.850	1.139	1.070	1.194	1.413*	2.940**	1.784*	4.110*	1.235
**Secondary**	2.415**	1.530**	1.445**	1.099	1.261*	1.865**	3.814**	2.033**	6.552**	1.909
**Primary**	3.262**	2.043**	1.774**	1.136*	1.018	1.164	2.017**	1.098	3.988*	1.458
**No schooling/ kindergarten**	4.736**	2.849**	1.745**	0.890	0.820*	0.624*	1.084	0.611	3.433*	1.561
**Number of observations**	18666	90501	268478	365885	269694	237841	198939	129437	45354	11066

** p<0.01.

* p<0.05.

**Fig 3:**
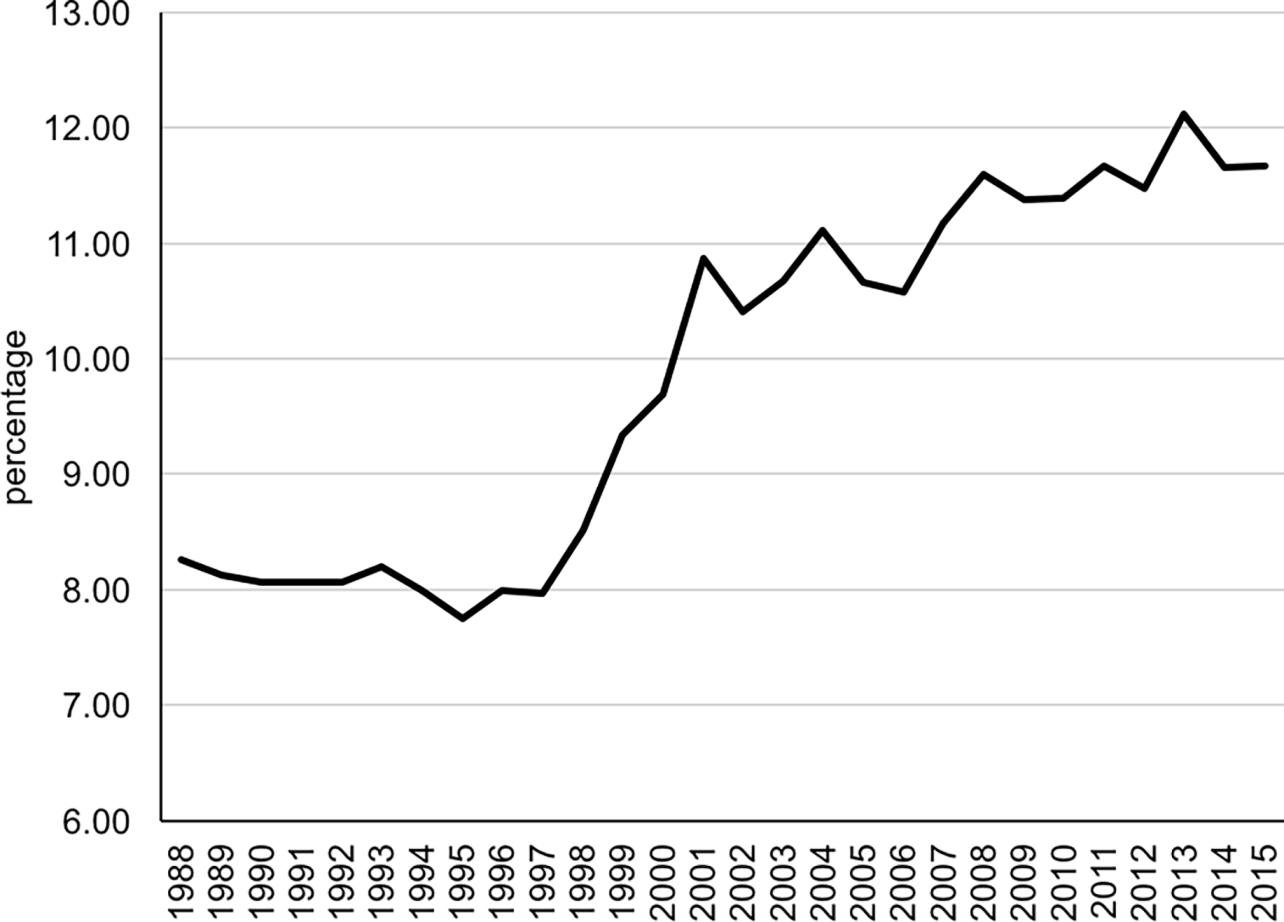
Prenuptial pregnancies by year of birth, Hong Kong (1988–2015).

**Fig 4:**
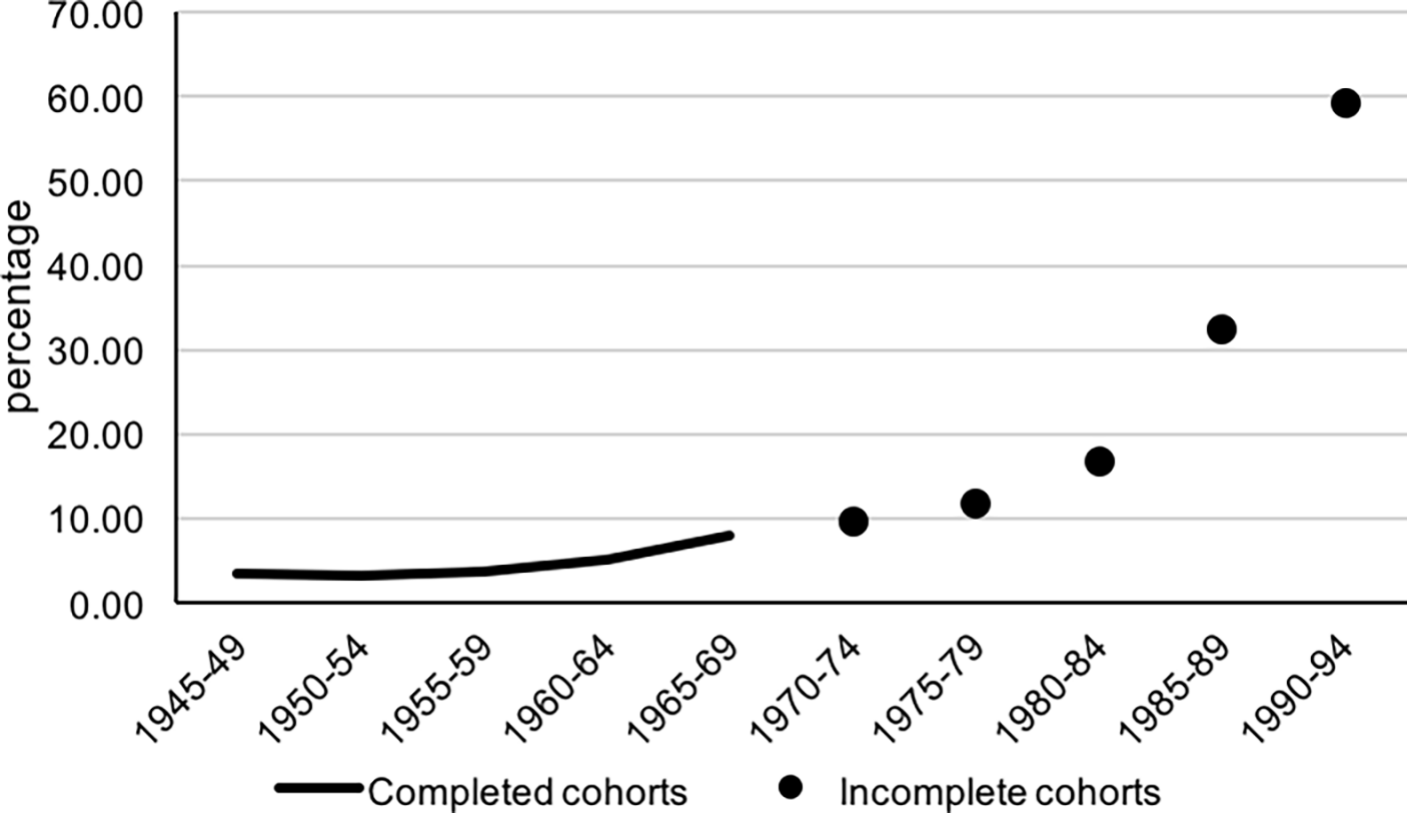
Prenuptial pregnancies by birth cohort of the mother, Hong Kong (1945–1994).

Fig 3 shows the percentage of PNP by year of birth of the child for the period 1988–2015. This percentage was fairly constant over 1988–1998, around 8%, but increased somewhat thereafter, reaching 11% by 2001 while it remained fairly constant in the period 2001–2015. Trends based on PNP considering births within 6 months of marriage are very similar though levels are reduced by about 2 percentage points on average. Regarding trends by birth cohort of the mother (Fig 4) the percentage of PNP based on births occurring within 8 months of marriage was quite low, 3–4%, among women born before 1960. Then there was a slight increase, reaching 8% for the 1965–69 cohort and 10% for the 1970–74 cohort, and a sharper increase thereafter. The very high proportions, over 50%, observed for women born in 1990 or later are likely linked, at least partly, to the selectivity of women giving birth at very young ages. Again, trends based on PNP considering births within six months of marriage are virtually identical, though levels are slightly lower.

Table 3 shows descriptive statistics for women having a PNP in comparison to women having a non-PNP birth within marriage. Among cohorts born before 1960 mean age of mother at birth is very similar for both groups. For women born thereafter, mean age at birth of those having a PNP is higher; the difference for the 1965–74 cohorts, who have more or less completed their reproductive career, is about 3.5–4 years. The vast majority of women having a PNP have secondary educational qualifications. On the other hand, higher proportions of women having a non-PNP have tertiary educational qualifications while they are also over-represented in the group that has completed only primary education or less.

**Table 3:** Descriptive statistics by birth cohort of the mother for women with or without a prenuptial birth: Hong Kong.

						*INCOMPLETE BIRTH COHORTS*				
	<1950	1950–54	1955–59	1960–64	1965–69	1970–74	1975–79	1980–84	1985–89	1990+
**Women having a PNP**^a^										
**Mean age at birth**	40.96	36.86	33.35	30.90	30.40	31.51	31.56	29.76	26.37	22.37
**Maternal education (%)**										
**Tertiary degree**	5.00	6.25	4.79	2.98	1.32	0.32	0.14	0.04	0.05	0.02
**Tertiary non-degree**	2.50	5.36	6.45	6.48	4.37	3.64	3.30	1.62	0.93	0.73
**Secondary**	55.83	53.87	64.06	71.89	79.72	80.32	77.28	75.82	76.28	84.58
**Primary**	34.17	31.65	22.70	14.72	8.49	6.08	6.28	8.23	10.63	9.44
**No schooling/kindergarten**	2.50	2.88	2.01	3.93	6.09	9.64	12.99	14.28	12.10	5.23
**Women not having a PNP**	40.98	37.19	33.14	29.51	26.97	26.47	26.72	25.99	24.26	21.16
**Mean age at birth**										
**Maternal education (%)**										
**Tertiary degree**	6.28	6.22	4.98	2.72	1.02	0.42	0.31	0.18	0.25	0.23
**Tertiary non-degree**	4.09	6.40	7.92	7.44	6.27	6.27	3.99	2.16	2.29	2.42
**Secondary**	47.13	53.43	60.24	69.02	70.54	61.36	54.43	55.12	65.94	81.67
**Primary**	37.20	31.01	23.75	15.34	10.06	8.55	9.61	11.46	11.86	10.17
**No schooling/kindergarten**	5.30	2.95	3.11	5.48	12.12	23.40	31.65	31.07	19.67	5.51

^**a**^ PNP is defined as a birth occurring within eight months of marriage.

Table 4 shows Odds Ratios (ORs) based on logistic regression models exploring predictors of PNP. With the exception of women born before 1955, younger age of the mother at the birth is linked to significantly higher chances of a PNP while controlling for year of birth and other characteristics of the mother. Lower maternal educational attainment among women born before 1970 seems to be inversely related to chances of a PNP. However, the opposite holds for younger cohorts; among women born since 1970 chances of such a pregnancy increase substantially for those with secondary, primary or fewer educational qualifications compared to women having obtained a tertiary degree. Findings based on PNPs defined as births occurring within 6 months of marriage (not presented here) are fairly similar, implying that the definition used in the analysis (births occurring within eight months of marriage) is robust.

**Table 4:** Odds Ratios based on Logistic Regression Models assessing predictors of prenuptial^a^ births by birth cohort of the mother: Hong Kong.

						*INCOMPLETE BIRTH COHORTS*				
	<1950	1950–54	1955–59	1960–64	1965–69	1970–74	1975–79	1980–84	1985–89	1990+
**Predictors**										
**Year of birth**	0.954	0.985	1.017	1.061**	1.062**	1.070**	1.041**	1.010	0.986	1.009
**Age of the mother at birth**	1.033	1.072**	0.967**	0.855**	0.798**	0.780**	0.781**	0.752**	0.742**	0.707**
**Maternal education (ref cat: Tertiary degree)**										
**Tertiary non-degree**	0.779	0.779	0.868	0.943	0.786**	1.025	2.579**	3.933**	1.980	3.750
**Secondary**	1.492	1.017	1.102	0.943	0.986	2.509**	7.211**	15.819**	10.069**	15.277*
**Primary**	1.146	1.039	0.984	0.804**	0.670**	1.819**	6.533**	14.426**	10.186**	17.064**
**No schooling/ kindergarten**	0.579	0.949	0.692**	0.883	0.904	1.957**	4.781**	11.315**	9.469**	23.154**
**Number of observations**	3370	30835	139319	279817	244556	219712	182315	117337	38772	7507

Note: The analysis refers to births occurring since 1988 as the relevant information to identify prenuptial births for earlier years is unavailable.

** p<0.01.

* p<0.05.

^**a**^ PNP is defined as a birth occurring within 8 months of marriage.

## Discussion

Our findings shon that, in common with elsewhere in East Asia, the percentage of BoM in Hong Kong is relatively low by OECD standards, and that the year-on-year trend shows only a modest increase. When measured by cohorts, the increase does appear to be much more striking. However, we should be very wary of interpreting the evidence derived from the youngest cohorts because of their incomplete childbearing careers. In particular, the fact that our modelling exercise suggests that younger women are more likely to have BoM coupled with the common observation that postponement of fertility in East Asia is strong, especially for women with tertiary education [15,42], leads us to suggest that upon completion of these cohorts we would expect, *ceteris paribus*, the percentage of women with BoM to decline from the figures presented here, and a much slower increasing overall trend.

The evidence relating to prenuptial pregnancies showed that, as is the case in Japan, prenuptial pregnancies appear to becoming a more significant ‘pathway’ towards childbearing in Hong Kong. Again, though, the usual caveats apply regarding interpreting the most recent, incomplete cohorts given that the women covered in this portion of the data are the most likely to have such pathways to childbearing. Despite this, it appears that prenuptial pregnancy seems to be an increasing ‘third way’ into family formation in Hong Kong, as in Japan. Further research is required to understand the decision-making process which translates pregnancy into marriage and birth rather than abortion in the case of Hong Kong [31]. In other words, are such unions ‘shotgun marriages’, or rather does pregnancy alter the temporal flow, ‘speeding up’ what would have happened anyway?

Taking into account these trends in BoM and prenuptial pregnancy from a period perspective, as well as with the caveats regarding the incomplete cohorts, we might suggest that under a business-as-usual scenario, the future trends may be likely to remain relatively low. Returning to the observation by other demographers for East Asia stated earlier [22], and Yip [21] for Hong Kong in particular, this may therefore serve to reinforce the very low fertility rates we see in the territory.

This view, however, takes a rather static view of the future. Of course, both marriage as an institution can change; as can attitudes of the population towards marriage and childbearing. It appears intuitively unlikely that there will be a return to ‘old-fashioned’ domestic ideals of gendered roles in the near future–a view confirmed by a recent survey of population experts [48,49]. While it is certainly the case that both young men and women favour a transformation in gender roles at home as stated in surveys [37,38,50], the reality is that the work culture and traditional attitudes may conspire to slow institutional changes [17,25]. As women’s educational and labour market opportunities continue to transform, the opportunity cost of such an unreformed ‘marriage package’ (to include childbearing) simply grows higher and higher. As such, without major reform to ‘what it is’, marriage can become ever more unattractive.

In this context, an international comparison via Second Demographic Transition Theory is valuable. Aspects of the Theory suggest that we might expect men and women to ‘rebel’ against this traditional view of marriage and childbearing, and ‘strike out’ on their own as ‘king couple’ to cohabit long-term, and bear children outside of marriage. More particularly, we might expect highly educated men and women, who are at the highest end of Maslowian drift and with the highest opportunity costs of entering into the unreformed ‘marriage package’ to be the ‘vanguard’ population in terms of driving and shaping new family forms. However, our analysis shows that, if anything, the opposite appears to be the case. Applying this finding to the future, we might suggest that as educational attainment levels for women (and men) increase, if such conservative attitudes concerning marriage prevail, then it is feasible that low rates of BoM could continue into the future.

Policies which might change this future pathway might include, firstly, the further reform of work culture, family support, childcare and other interventions to lower the actual and opportunity cost of family formation, thus making the transition to marriage and childbearing less of a rupture in life and career. Secondly, other policies which equalise the benefits of marrying over cohabiting may also serve to affect future attitudes regarding the relationship between birth and marriage. However, such policies are likely to be unpopular with many groups in society, especially those with a conservative view of the family.

Our analysis showed that prevalence of BoM and prenuptial pregnancy are not evenly distributed by education. Rather, there appears to be a bifurcation between secondary and tertiary educated women. While we have explored the possible reasons for this above, there are likely to be some clear policy implications from this finding. As we identified in the introduction, there is still an expected link between childbearing and marriage. Families which do not conform to this norm are privy to many fewer rights and privileges [34–35]. In this context, there is a risk that such families characterised by lower education attainment who may already be more economically vulnerable may suffer a double vulnerability through the marital status in relation to their offspring. Firstly, although the evidence presented in the study suggests that the vast majority of BoM are born to cohabiting couples rather than to the classically defined, and oft-stigmatised ‘single mum’, it is essential to ensure children born under such conditions are not penalised. Ensuring this without being seen to be ‘encouraging’ BoM and, thus, offending local cultural and political sensibilities is, undoubtedly, a significant challenge for policymakers. Secondly, there has been little research on the extent to which PNPs are stigmatised in Hong Kong society–either at the time of marriage or beyond. Having said this, the bifurcation of experience relating to BoM and prenuptial pregnancies suggest that there is still a role for family planning activities in Hong Kong; and that these activities may be targeted more especially at couples with secondary educational attainment.

While we have employed micro-level data, a weakness of our analysis is that we have generally presented only aggregated, cross-sectional evidence of trends and predictors. In order to develop a deeper, and more nuanced understanding of the relationship between marriage and childbearing, it may be necessary to explore individual, longitudinal life-course pathways. This would allow us, for example, to examine whether marriage occurs after a first BoM, or whether cohabiting partnerships are stable over long periods of time. Such an exercise requires a complex procedure of probabilistic longitudinal matching of various datasets over time, which is beyond the scope of this paper. Finally, further analysis could explore the *spatial* dynamics of such BoM in order to better target family planning services and resources.
